# Adipocyte-derived relaxing factor—a fat load to be learnt

**DOI:** 10.1093/ejcts/ezac120

**Published:** 2022-02-25

**Authors:** Amer Harky, Jeffrey Shi Kai Chan, Jeremy Man Ho Hui, Gopal Soppa

**Affiliations:** 1 Department of Cardiothoracic Surgery, Liverpool Heart and Lung Hospital, Liverpool, UK; 2 Cardiovascular Analytics Group, UK–Hong Kong–China Collaboration, China; 3 Division of Cardiology, Department of Medicine and Therapeutics, Prince of Wales Hospital, Hong Kong, China; 4 Department of Medicine, The University of Hong Kong, Hong Kong, China

**Keywords:** perivascular tissue, PVAT, ADRF, adipocyte-derived relaing factor, radial artery, coronary artery bypass graft

## Abstract

Properties of the radial artery (RA) have been of immense interest to both laboratory scientists and clinicians, especially due to the increasing utilization of RA as a second-choice conduit for coronary artery bypass graft (CABG), after left internal mammary artery.

Properties of the radial artery (RA) have been of immense interest to both laboratory scientists and clinicians, especially due to the increasing utilization of RA as a second-choice conduit for coronary artery bypass graft (CABG), after left internal mammary artery. In the current study, Kociszewska *et al.* [1] ought to be congratulated for performing 4 elegant experiments that demonstrated important anticontractile properties of the perivascular tissue (PVAT) in RA, which was likely mediated by the adipocyte-derived relaxing factor (ADRF). 

Clinically, the findings by Kociszewska *et al.* further support the use of pedicle, instead of skeletonized RA grafts in CABG. Despite initial enthusiasm in using skeletonized RA grafts with sporadic reports of improved outcomes [2], later reports have not shown significant differences in clinical outcomes between skeletonized and pedicle RA conduits [3]. Nonetheless, no study has been done to specifically look at vasospasm as an outcome [3], despite prior laboratory studies having looked at vasospasm. The findings of the current study strongly suggest that retaining the PVAT during RA harvesting may mitigate the risk of vasospasm which is well known for RA grafts. This is in addition to the traditional reasoning of minimizing manipulation and thus the risk of endothelial damage during harvesting by retaining a pedicle instead of skeletonizing the graft. Overall, the findings agreed with the recommendations set forth in the 2018 European Society of Cardiology/European Association for Cardio-Thoracic Surgery guidelines for myocardial revascularization [4], which recommended harvesting of the entire RA pedicle in order to prevent vasospasm.

Interestingly, the authors demonstrated that ADRF could not have exerted its anticontractile effects via potassium channels, at least not as a dominant mechanism. This contrasted with prior observations made with the human internal mammary artery (IMA) PVAT, suggesting that vascular physiology, even in similar types of vessels, likely differs significantly between different anatomical sites. Another such difference may be in the activity of the autonomic nervous system in PVAT, which has been extensively investigated in non-RA PVAT. Ayala-Lopez *et al.* [5] showed that catecholamines, including dopamine, norepinephrine and adrenaline, were present in PVAT. It was shown that tyramine, a sympathomimetic, induced contraction in rat thoracic aorta and superior mesenteric artery in a PVAT- and concentration-dependent manner, which was blunted by a norepinephrine reuptake inhibition and vesicular monoamine transporter blockade, and abolished by α_1_ adrenergic blockade, indicating that tyramine stimulates catecholamine release from PVAT. More importantly, the cytoplasm of adipocytes in PVAT contained norepinephrine, and removal of the coeliac ganglion, which supplies catecholamines to the superior mesenteric artery PVAT, did not reduce its tyramine-induced contraction. This implies that PVAT can release catecholamines independently of the sympathetic nervous system to cause arterial contraction. In addition, PVAT has a norepinephrine uptake mechanism in mesenteric arteries, likely mediated by the organic cation transporter 3 (OCT3), that reduces vasoconstriction [6]. The uptake of norepinephrine in mesenteric PVAT was reduced by inhibition of the norepinephrine transporter, the serotonin transporter and OCT3, but only OCT3 mRNA and protein in PVAT adipocytes were detected. While the present study does not completely exclude the possibility that a norepinephrine uptake system may modulate the vasorelaxant properties of RA PVAT, such system is unlikely to be active in extracted aliquots, indicating that it is unlikely to play a dominant role in the anticontractile functions of RA PVAT.

Despite the observed differences between RA and IMA in the factors mediating ADRF’s anticontractile effects, a 2019 pairwise and network meta-analysis of 15 clinical studies did not observe any significant difference in clinical outcomes between using RA and IMA as arterial conduits for CABG [7]. The anticontractile properties of the RA PVAT, as well as the association between pedicled IMA use and deep sternal wound infections [7], may be arguments in favour of increased use of pedicled RA as an arterial conduit. The current study’s findings also raise the possibility of identifying specific post-CABG anti-spasmodic agents for different arterial conduits. Despite the various anti-spasmodic agents available, there is no consensus on the optimal agent for post-CABG use. A single-centre pilot randomized controlled trial is currently underway to compare the efficacy of 3 different anti-spasmodic agents (NCT04310995) and is anticipated to be completed by the end of 2022. It will be interesting to relate the results of this trial to the findings of the current study.

Meanwhile, previous studies on rat aorta have suggested that hydrogen sulphide may mediate the effects of ADRF [8]. This was not specifically investigated in the present study. Given the possible site-specific nature of ADRF’s properties, the above roles of hydrogen sulphide in ADRF effects cannot be assumed to be generalizable to ADRF from RA PVAT, and future investigations should be considered for investigating its role specifically. The current study was also limited by the significant heterogeneity in clinical states of patients and histological structures of analysed PVAT, as rightly acknowledged by the authors. Differences in adipocyte compositions of PVAT may have important effects. Multiple studies have found white-like PVAT, with a domination of white adipose tissue, to be accompanied by a loss of anticontractile effects [9, 10]. Such heterogeneity and the small sample size (*N* = 15) in the current study raise questions about the generalizability of the findings, and whether the results are extrapolatable to wider, clinically more relevant populations remains to be seen. 

In summary, Kociszewska *et al.* are to be congratulated for their important contribution to the understanding of RA PVAT physiology. While reinforcing current clinical guidelines’ recommendations, much remains to be learnt both in the laboratory and clinically, with the potential of bringing exciting changes to the field.
